# Increased functional sensorimotor network efficiency relates to disability in multiple sclerosis

**DOI:** 10.1177/1352458520966292

**Published:** 2020-10-26

**Authors:** Myrte Strik, Declan T Chard, Iris Dekker, Kim A Meijer, Anand JC Eijlers, Matteo Pardini, Bernard MJ Uitdehaag, Scott C Kolbe, Jeroen JG Geurts, Menno M Schoonheim

**Affiliations:** Department of Anatomy and Neurosciences, MS Center Amsterdam, Amsterdam Neuroscience, Amsterdam UMC, Vrije Universiteit Amsterdam, Amsterdam, The Netherlands/Department of Radiology and Medicine, The University of Melbourne, Melbourne, VIC, Australia; NMR Research Unit, Queen Square MS Centre, Department of Neuroinflammation, UCL Institute of Neurology, London, UK/National Institute for Health Research, University College London Hospitals Biomedical Research Centre, London, UK; Department of Neurology, MS Center Amsterdam, Amsterdam Neuroscience, Amsterdam UMC, Vrije Universiteit Amsterdam, Amsterdam, The Netherlands/Department of Radiology and Nuclear Medicine, MS Center Amsterdam, Amsterdam Neuroscience, Amsterdam UMC, Vrije Universiteit Amsterdam, Amsterdam, The Netherlands; Department of Anatomy and Neurosciences, MS Center Amsterdam, Amsterdam Neuroscience, Amsterdam UMC, Vrije Universiteit Amsterdam, Amsterdam, The Netherlands; Department of Anatomy and Neurosciences, MS Center Amsterdam, Amsterdam Neuroscience, Amsterdam UMC, Vrije Universiteit Amsterdam, Amsterdam, The Netherlands; NMR Research Unit, Queen Square MS Centre, Department of Neuroinflammation, UCL Institute of Neurology, London, UK/Department of Neurosciences, Rehabilitation, Ophthalmology, Genetics and Maternal and Child Health, University of Genoa, Genoa, Italy/Ospedale Policlinico San Martino-IRCCS, Genoa, Italy; Department of Neurology, MS Center Amsterdam, Amsterdam Neuroscience, Amsterdam UMC, Vrije Universiteit Amsterdam, Amsterdam, The Netherlands; Department of Radiology and Medicine, The University of Melbourne, Melbourne, VIC, Australia/Department of Neurosciences, Central Clinical School, Monash University, Melbourne, VIC, Australia; Department of Anatomy and Neurosciences, MS Center Amsterdam, Amsterdam Neuroscience, Amsterdam UMC, Vrije Universiteit Amsterdam, Amsterdam, The Netherlands; Department of Anatomy and Neurosciences, MS Center Amsterdam, Amsterdam Neuroscience, Amsterdam UMC, Vrije Universiteit Amsterdam, Amsterdam, The Netherlands

**Keywords:** Multiple sclerosis, resting-state, functional MRI, disability, network, efficiency

## Abstract

**Background:**

Network abnormalities could help explain physical disability in multiple sclerosis (MS), which remains poorly understood.

**Objective:**

This study investigates functional network efficiency changes in the sensorimotor system.

**Methods:**

We included 222 MS patients, divided into low disability (LD, Expanded Disability Status Scale (EDSS) ⩽3.5, *n* = 185) and high disability (HD, EDSS ⩾6, *n* = 37), and 82 healthy controls (HC). Functional connectivity was assessed between 23 sensorimotor regions. Measures of efficiency were computed and compared between groups using general linear models corrected for age and sex. Binary logistic regression models related disability status to local functional network efficiency (LE), brain volumes and demographics. Functional connectivity patterns of regions important for disability were explored.

**Results:**

HD patients demonstrated significantly higher LE of the left primary somatosensory cortex (S1) and right pallidum compared to LD and HC, and left premotor cortex compared to HC only. The logistic regression model for disability (*R*^2^ = 0.38) included age, deep grey matter volume and left S1 LE. S1 functional connectivity was increased with prefrontal and secondary sensory areas in HD patients, compared to LD and HC.

**Conclusion:**

Clinical disability in MS associates with functional sensorimotor increases in efficiency and connectivity, centred around S1, independent of structural damage.

## Introduction

Multiple sclerosis (MS) is a demyelinating and neurodegenerative disorder of the central nervous system leading to disabling sensorimotor impairments.^1^ The pathophysiology of neurological dysfunction is complex and not fully understood. Conventional magnetic resonance imaging (MRI) measures of lesion load in brain and spinal cord have shown only modest relations with disability.^2,3^ More advanced measures, such as spinal cord^4^ and (sub)cortical grey matter (GM) atrophy,^5^ relate more strongly to disability, but still do not fully explain clinical heterogeneity, suggesting that additional processes may also be important determinants of disability.

In addition to structural brain changes, functional alterations might be relevant to disability progression. Resting-state functional MRI (fMRI) has been used to identify changes within sensorimotor network (SMN) from the earliest stages of MS. However, results have been difficult to interpret due to conflicting observations with increased^6–10^ and decreased^11–15^ functional connectivity (FC), local^14^ and widespread connectivity changes,^6,7,12^ and limited or conflicting evidence of clinical correlations.^6–9,11–15^ In part, differences between studies are likely to reflect differences in cohorts, and limited correlations with outcomes to reflect the inclusion of networks that are not relevant to clinical outcomes that have been assessed.

In this study, we assessed the motor system using the concept of network efficiency,^16^ a well-validated measure of the integration and segregation of information processing in the brain. We assessed functional efficiency in the presence or absence of overt motor disability in a large cohort and investigated the most important correlates of sensorimotor disability in MS.

## Methods

### Participants

For this study we included 222 patients (age 45.35 ± 10.47, 165 females (74%)) with MS (disease duration 13.60 ± 8.22), part of the Amsterdam MS Cohort,^17^ and 82 healthy controls (HC, age 45.93 ± 10.79, 52 females (63%)). Inclusion criteria included the presence of relapse-onset MS, availability of disability measurements, either high or low disability severity (see below) and whole-brain and cerebellar functional coverage. All patients included in this study were diagnosed with clinically definite MS according to the 2010 revised McDonald criteria^18^ and did not experience a relapse in the 2 months prior to the scanning session. This study was approved by the local institutional ethics review board and all participants provided written consent before participation.

### Motor disability assessments and group definition

Disability was assessed in patients using the Expanded Disability Status Scale (EDSS).^19^ We divided patients into two groups based on walking impairments; that is, low disability (LD, EDSS ⩽ 3.5 (no or minimal walking impairment), *n* = 185, age 43.83 ± 10.12, 135 females) and high disability (HD, EDSS ⩾ 6 (unable to walk without aid or assistance), *n* = 37, age 52.94 ± 8.87, 30 females). Subsequently, the low disability group was reduced into only patients with very minimal disability, EDSS ⩽ 2, after which general linear model (GLM) and regression analyses (see below) were repeated.

### Imaging data acquisition

All participants were scanned using a 3T-MRI (GE Signa HDxt, Milwaukee, WI) with an 8-channel phased-array head coil. Functional whole-brain resting-state MRI data were acquired with an echo planar imaging sequence (repetition time (TR) = 2200 ms, echo time (TE) = 35 ms, flip angle (FA) = 80°, 3 mm contiguous axial slices, in-plane resolution 3.3 × 3.3 mm^2^). For brain volumetric calculations, a three-dimensional (3D) T1-weighted fast spoiled gradient-echo sequence was used (TR = 7.8 ms, TE = 3.0 ms, FA = 12°, inversion time (TI) = 450 ms, 1.0 mm sagittal slices, 0.9 × 0.9 mm^2^ in-plane resolution), and a 3D fluid-attenuated inversion recovery (FLAIR) was acquired to identify white matter (WM) lesions (TR = 8000 ms, TE = 125 ms, TI = 2350 ms, 1.2 mm sagittal slices, 0.98 × 0.98 mm^2^ in-plane resolution).

### White matter lesion segmentation and brain volume calculations

WM lesions were automatically segmented on FLAIR images using k-nearest neighbour classification with tissue type priors.^20^ These images were registered to T1-weighted images and lesions were filled using Lesion Automated Pre-processing.^21^ Lesion-filled images were subsequently used to calculate total brain volumes and subcortical volumes using SIENAX and FIRST, respectively (https://fsl.fmrib.ox.ac.uk/fsl/). Deep GM (DGM) volumes were subtracted from the total GM volume to calculate cortical GM volume specifically. To account for differences in head size, all brain volumes were normalized using V-scaling factor derived from SIENAX.

### Resting-state fMRI pre-processing

Resting-state fMRI pre-processing involved removal of the first two volumes, brain extraction, head motion correction, spatial smoothing with a 5-mm full width at half-maximum Gaussian kernel and high-pass temporal filtering (100 seconds cut off) using the MELODIC pipeline (FSL5). Registration parameters were calculated between fMRI and 3DT1 sequences, using boundary-based registration (BBR), and between 3DT1 and the standard brain using non-linear registration, both of which were inverted to co-register regions of interest (ROIs) to the fMRI sequence (see below). Images were checked for head motion, artefacts and registration errors. Level of motion was calculated based on the average frame-to-frame head motion, as reported previously.^22^ The average frame-to-frame head motion did not exceed more than one voxel (3 mm) and did not differ between patients and HC (*p* = 0.36). In addition, voxels without reliable signal caused by echo-planar imaging distortions, artefacts or non-brain tissue were excluded using a robust range-based threshold.

### Regions of interest

The cortex was segmented using the Brainnetome atlas (http://atlas.brainnetome.org), the cerebellum using the Harvard-Oxford atlas and DGM structures using FIRST. All ROIs were combined to form one atlas, subsequently registered to individual functional scans using inverted BBR parameters and nearest-neighbour interpolation and assessed on sufficient reliable signal, that is, at least 30% of voxels in at least 90% of all subjects after removing unreliable voxels. This resulted in the removal of orbitofrontal, inferior temporal cortices and nucleus accumbens and a final atlas containing 193 ROIs. Signal intensities within each individual atlas region were averaged for each time point to form 193 time series and FC was calculated with Pearson correlations, generating a 193 by 193 weighted undirected connectivity matrix. People have very distinct FC profiles,^23^ for which we corrected by dividing each connection with average whole-brain FC, enabling us to find disease-related changes in network patterns with relative FC values (Supplementary Figure 1). All negative correlations were set to zero.^22^

### The sensorimotor system

**Figure 1.**
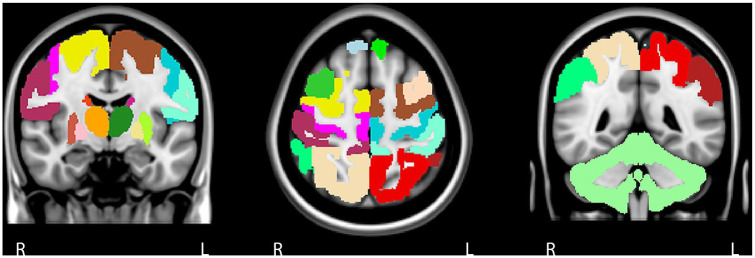
The sensorimotor network. The sensorimotor network was defined based on previous literature and we included 23 cortical and subcortical grey matter areas. The cortical components of the motor network are the primary motor cortex, premotor cortex, supplementary motor area, prefrontal cortex, primary somatosensory cortex, secondary sensory cortex and posterior associative sensory cortex. Each cortical area was subdivided into a right and left region of interest. As for subcortical regions, we included the cerebellum and left and right thalamus, caudate nucleus, putamen and pallidum.

We chose to include a broad range of regions beyond conventional ‘typical’ motor areas such as primary motor cortex (M1) and primary somatosensory cortex (S1), based on a previously published approach.^24^ This consisted of left and right frontal, parietal and cortical motor regions, DGM areas and cerebellum, forming a 23 × 23 FC matrix (Figure 1). The cerebellum was included as whole because of limited scan coverage, difficulties in parcellating only motor regions accurately on fMRI and to limit the total number of ROIs. Global efficiency (GE) of the entire SMN and local efficiency (LE) for each ROI was calculated using the Brain Connectivity Toolbox (https://sites.google.com/site/bctnet/). The global efficiency is based on the inverse of the average shortest path length of all individual network links, representing how efficient information flows throughout the entire SMN. The LE quantifies efficiency on a smaller scale and is related to the clustering coefficient, a network topology characteristic that reflects local processing of information.^16^

### Statistical data analyses

All statistical analyses were performed in SPSS 22 (IBM, USA). All measures were checked for normality using the Kolmogorov–Smirnov test and visual inspection of histograms. Lesion volumes were non-normally distributed and log-transformed. Non-normally distributed variables were compared with nonparametric tests (Mann–Whitney *U* test). For normally distributed variables, GLMs were used correcting sex and age with Bonferroni correction over groups; significance after correcting for the number of variables is also reported. To assess the most important correlates of disability, a binary logistic regression model with backward elimination was used including significant network measures, brain and lesion volumes and demographics. GLM and regression analyses repeated including LD patients (EDSS ⩽ 2) and HD patients. Areas where LE significantly contributed to the regression model were further explored by examining FC to other sensorimotor areas between groups using GLMs and Spearman correlations were performed between LE and functional system scores (FSS).

## Results

### Demographics, clinical data and brain volumes

Demographics, clinical variables and brain volumes are shown in Table 1. The highly disabled group had a significant longer disease duration (*p* < 0.001) and consisted of relatively more secondary progressive MS patients (*p* < 0.001) than the group with low disability. HD patients were significantly older compared to both LD patients (*p* < 0.001) and HC (*p* < 0.01). No differences in handedness were found between patients with high and low disability.

**Table 1.** Demographics, clinical and MRI characteristics.

	**Healthy controls**	**Low disability**	**High disability**
**Demographics**			
• Sex, F/M	52/30	135/50	30/7
• Age, years	45.93 (10.79)	43.83 (10.12)	52.94 (8.87)^a,b^
• Disease duration		12.07 (7.27)	21.23 (8.55)^b^
• Phenotypes (RRMS/SPMS), *n*		178/7	11/26^b^
• EDSS Total^c^		2.5 (0–3.5)	6.5 (6–8)^b^
• FSS Cerebellar^c^		1 (0–3)	3 (0–4)^b^
• FSS Pyramidal^c^		1 (0–3)	3 (2–5)^b^
• FSS Sensory^c^		2 (0–3)	2.5 (1–5)^b^
• FSS Brainstem^c^		0 (0–3)	1 (0–3)^b^
• FSS Visual^c^		0 (0–3)	1 (0–5)^b^
• FSS Bowel and Bladder^c^		0 (0–3)	2 (0–4)^b^
• FSS Cerebral (mental)^c^		1 (0–3)	2 (0–3)^b^
**Brain volumes**			
• NBV, L	1.51 (0.07)	1.48 (0.07)^a^	1.40 (0.07)^a,b^
• NWMV, L	0.69 (0.03)	0.67 (0.03)^a^	0.65 (0.03)^a,b^
• NCGMV, L	0.78 (0.05)	0.77 (0.05)^a^	0.72 (0.06)^a,b^
• NDGMV, mL	62.85 (3.75)	58.31 (5.80)^a^	51.71 (7.10)^a,b^
• Lesion volume (log), mL		3.88 (0.38)	4.25 (0.40)^b^

MRI: magnetic resonance imaging; F: female; M: male; RRMS: relapsing-remitting multiple sclerosis; SPMS: secondary progressive multiple sclerosis; EDSS: Expanded Disability Status Scale; FSS: Functional Systems Scores; NBV: normalized brain volume; NWMV: normalized white matter volume; NCGMV: normalized cortical grey matter volume; NDGMV: normalized deep grey matter volume.

All values represent means and standard deviations unless denotes otherwise.

a Significant difference compared to healthy controls.

b Significant difference compared to patients with low disability.

c Median and range.

Compared to HC, both MS groups displayed significantly lower whole brain, WM, cortical and DGM volumes (*p* < 0.05). HD patients exhibited more pronounced cortical GM atrophy (*p* = 0.001), DGM atrophy (*p* < 0.001), and whole brain (*p* < 0.001) and WM volume loss (*p* < 0.05) compared to LD patients. In addition, HD patients demonstrated a higher lesion volume compared to LD patients (*p* < 0.001).

### Global and local efficiency of the SMN

Global efficiency was not statistically significant. Local efficiency (see Table 2) was higher in the HD group compared to HC in left premotor cortex (*p* = 0.011), S1 (*p* = 0.001) and right pallidum (*p* = 0.044). HD patients showed higher LE in left S1 (*p* = 0.013) and right pallidum (*p* = 0.040) compared to LD patients. No differences in efficiency were seen between LD patients and controls. The LE of S1 remains significant after also correcting for the number of variables. These comparisons were not significant when using uncorrected FC matrices.

**Table 2.** The mean local efficiency values of each sensorimotor region for each group.

Sensorimotor regions	HC	LD	HD	*p*-values
Left prefrontal cortex	0.95 (0.11)	0.96 (0.12)	1.00 (0.13)	0.059
Left supplementary motor area	1.09 (0.16)	1.11 (0.20)	1.16 (0.28)	0.391
**Left premotor cortex**	**1.04 (0.15)**	**1.07 (0.18)**	**1.16 (0.28)** ^a^	**0.015**
Left primary motor cortex	1.05 (0.14)	1.08 (0.16)	1.14 (0.20)	0.099
**Left primary somatosensory cortex**	**1.07 (0.12)**	**1.09 (0.16)**	**1.20 (0.23)** ^a,b^	**0.002**
Left secondary sensory cortex	1.06 (0.12)	1.08 (0.14)	1.15 (0.24)	0.051
Left posterior associative sensory cortex	1.07 (0.14)	1.09 (0.16)	1.16 (0.23)	0.172
Left thalamus	1.08 (0.23)	1.11 (0.24)	1.20 (0.37)	0.352
Left caudate nucleus	1.05 (0.21)	1.03 (0.25)	1.12 (0.29)	0.272
Left putamen	1.07 (0.19)	1.05 (0.22)	1.14 (0.28)	0.297
Left pallidum	0.96 (0.20)	0.95 (0.20)	1.08 (0.41)	0.105
Right prefrontal cortex	0.95 (0.16)	0.97 (0.12)	1.01 (0.15)	0.084
Right supplementary motor area	1.10 (0.17)	1.12 (0.19)	1.16 (0.24)	0.556
Right premotor cortex	1.06 (0.15)	1.07 (0.17)	1.14 (0.23)	0.269
Right primary motor cortex	1.05 (0.14)	1.08 (0.16)	1.13 (0.19)	0.188
Right primary somatosensory cortex	1.08 (0.13)	1.10 (0.17)	1.18 (0.21)	0.064
Right secondary sensory cortex	1.07 (0.15)	1.07 (0.15)	1.14 (0.20)	0.395
Right posterior associative sensory cortex	1.07 (0.13)	1.08 (0.17)	1.15 (0.24)	0.257
Right thalamus	1.08 (0.22)	1.10 (0.25)	1.20 (0.34)	0.384
Right caudate nucleus	1.06 (0.20)	1.06 (0.23)	1.11 (0.36)	0.747
Right putamen	1.07 (0.20)	1.06 (0.24)	1.14 (0.27)	0.453
**Right pallidum**	**0.95 (0.18)**	**0.96 (0.22)**	**1.10 (0.46)** ^a,b^	**0.033**
Cerebellum	1.02 (0.20)	1.05 (0.21)	0.96 (0.23)	0.095

HC: healthy controls; LD: patients with low disability; HD: patients with high disability.

General linear model was used to compare local efficiency differences between groups and main effects were significant for the left premotor cortex, left primary somatosensory cortex and right pallidum (in bold).

a After post hoc Bonferroni correction, significant differences were found compared to healthy controls.

b After post hoc Bonferroni correction, significant differences were found compared to patients with low disability.

In addition, when compared to the low disability group based on EDSS ⩽ 2, highly disabled patients showed higher GE (*p* = 0.026) and LE of the left premotor cortex (*p* = 0.035), M1 (*p* = 0.011), S1 (*p* = 0.001), pallidum (*p* = 0.016) and right M1 (*p* = 0.033), S1 (*p* = 0.007), putamen (*p* = 0.040) and pallidum (*p* = 0.005). Also here, S1 remains significant after additional correction.

### Predictors of disability in MS

A final backward binary logistic regression model was created to identify the most important correlates of higher disability (Nagelkerke *R*^2^ = 0.36, chi-square = 53.56, *p* < 0.001), which included a higher age (Wald = 9.71, *p* = 0.002), lower DGM volume (Wald = 22.19, *p* < 0.001) and higher LE of left S1 (Wald = 6.26, *p* = 0.012). Repeating this regression model using only right-handed patients did not change results. In addition, when repeating this regression model after redefining ‘low disability’ to EDSS ⩽ 2, the same predictors were identified.

### Changes in primary somatosensory FC

**Figure 2.**
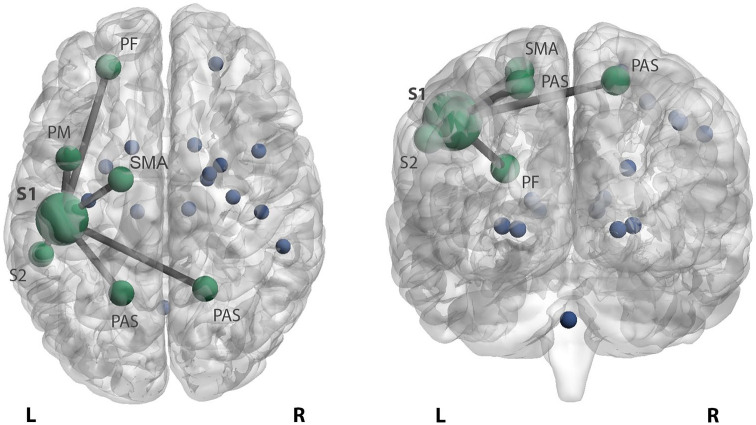
Increased functional connectivity of the somatosensory cortex in highly disabled MS patients. Patients with high disability display higher primary left somatosensory connectivity with the left prefrontal cortex (PF), premotor cortex (PM), secondary sensory cortex (S2) and right and left posterior associative sensory cortex (PAS) compared to patients with low disability and HC. In addition, stronger connectivity with the left supplementary motor area (SMA) was seen in highly disabled patients compared to HC. The stronger connectivity between these areas is reflected by edges between the bigger nodes in green. The blue dots reflect the remaining sensorimotor network regions. L: left; R: right.

HD patients displayed significantly higher FC between left S1 and left prefrontal cortex (*p* = 0.001 vs HC and *p* = 0.004 vs LD), premotor cortex (*p* < 0.001 and *p* = 0.023), secondary sensory cortex (*p* = 0.002 and *p* = 0.011) and right (*p* = 0.002 and *p* = 0.026) and left (*p* < 0.001 and *p* = 0.007) posterior associative sensory cortex compared to HC and LD patients (Figure 2). In addition, compared only to HC, HD patients displayed higher left S1 connectivity with left supplementary motor area (SMA) (*p* = 0.031). In patients with LD, no connectivity changes of the left S1 cortex were found compared to HC. After additional correction, prefrontal, premotor and posterior associative sensory cortices remained significant.

### Relations between left S1 changes and clinical functional subsystems

**Figure 3.**
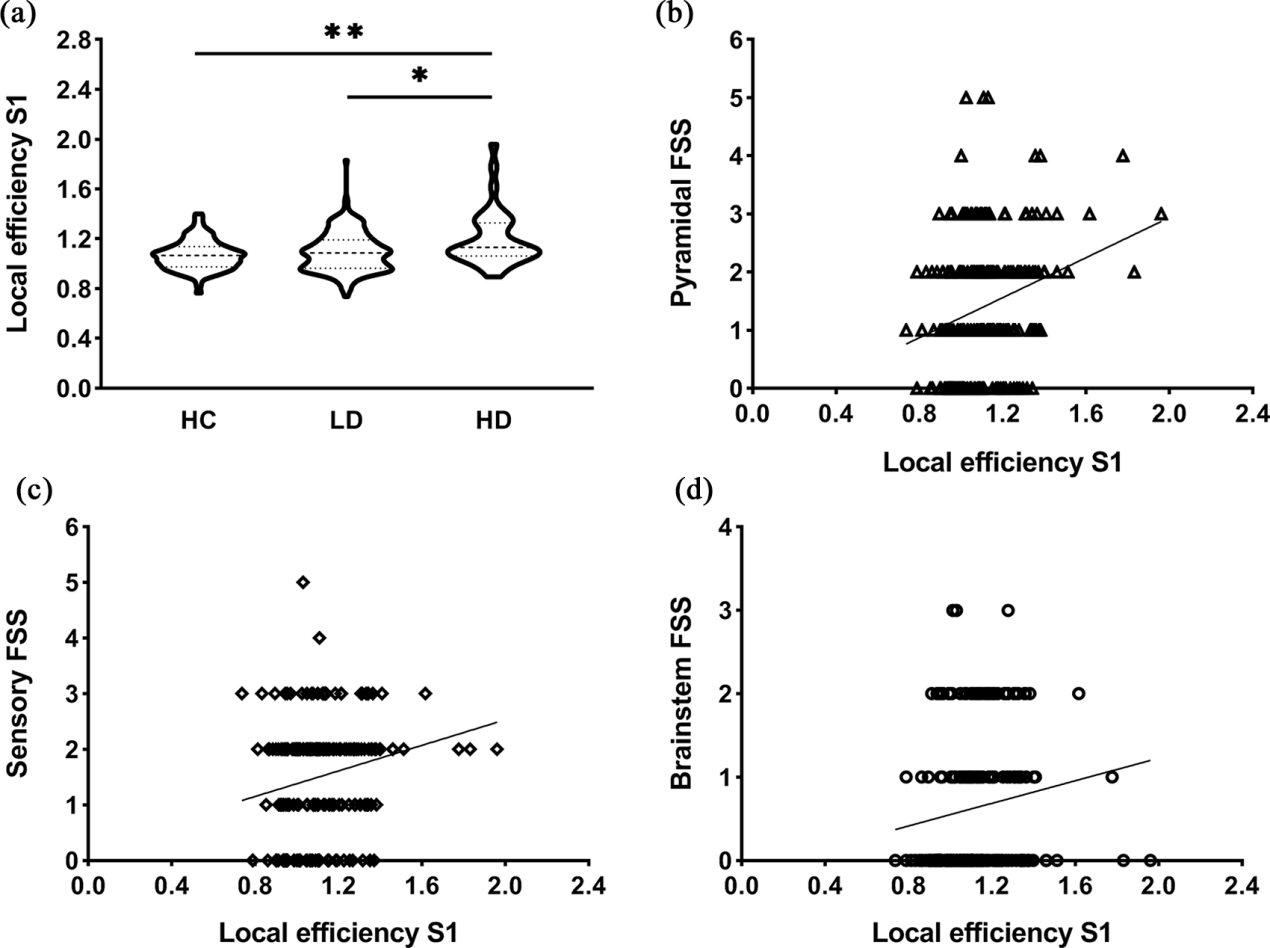
Efficiency of the left primary somatosensory cortex and relations with functional systems scores. (a) Highly disabled MS patients demonstrated significantly higher local efficiency (LE) of S1 compared to both patients with low disability (*p < 0.05) and control subjects (**p < 0.01). The violin plots show the data distributions, that is, the kernel density reflects an estimation of underlying distributed data, and the dashed lines represent the median and the quartiles. (b–d) The scatter plots display significant correlations between LE of left S1 and clinical functional system scores including pyramidal (*r* = 0.239, *p* < 0.001) (b), sensory (*r* = 0.211, *p* = 0.002) (c) and brainstem (*r* = 0.210, *p* = 0.002) (d). Higher LE of S1 significantly correlated with worse pyramidal, brainstem and sensory FSS. HC: healthy controls; LD: patients with low disability; HD: highly disabled patients; S1: primary somatosensory cortex; FSS: functional system scores.

In MS, higher S1 LE significantly correlated with worse pyramidal (*r* = 0.239, *p* < 0.001), brainstem (*r* = 0.210, *p* = 0.002), sensory (*r* = 0.211, *p* = 0.002) FSS and EDSS (*r* = 0.256, *p* < 0.001) (Figure 3).

## Discussion

In this study, we investigated whether FC and efficiency within the SMN could explain disability in MS and whether specific regions might be particularly affected. Patients with high disability showed extensive SMN changes, mostly centred around S1 as well as the premotor area and pallidum. Patients with minimal disability showed no changes. Increased network efficiency and connectivity of S1 was related to worse disability, even after correcting for structural damage.

### S1 and disability in MS

We explicitly identified network changes of S1 that correlated with disability and worse sensory, pyramidal and brainstem FSS (see Figure 3). Impairments in S1 function have shown to be a significant contributor to ineffective motor output and function in other neurological conditions like stroke,^25^ but in MS this has not been demonstrated previously. Previous MS literature investigating functional changes has been heterogeneous, identifying global changes that were typically not directly related to disability.^6–8,12,14^ One of the first fMRI studies looking at the sensorimotor system investigated connectivity of M1 and showed decreased interhemispheric connectivity but without any clinical correlates.^14^ Instead of one specific area, more recent studies investigated the sensorimotor system using independent component analyses (ICA) and reported either only subcortical changes^8^ or global alterations,^6,7,12^ but again no significant clinical relations were found.

Previous literature did not clearly link SMN changes to clinical scores, which could possibly be explained by the common exclusion of cerebellar, subcortical and prefrontal GM structures. These areas were often not included by ICA as ‘motor network regions’, but considered as a separate network^10,11,13^ or part of other networks investigated,^6,12^ despite their critical role in sensorimotor functioning and processing. This omission of previous sensorimotor connectivity findings within the framework of motor disability may therefore be the result of an overly simplistic view of network functioning, which may be counteracted by applying whole-network measures such as efficiency. Nonetheless, a connectivity approach was valuable post hoc, as seen in our data, to show that secondary processing areas, that is, prefrontal cortex, premotor cortex, SMA and secondary sensory cortex, had higher connectivity with S1 (Figure 2), the primary predictor of disability in MS, while primary motor areas (such as M1) did not. These supplementary motor areas play an essential role in the fine balance between somatosensory processing and motor production and altered cortical connectivity might therefore reflect disturbed sensorimotor processing.

### Complex patterns of increases versus decreases

Overall, both efficiency and connectivity measures in our study showed increases in disabled patients compared to those with lower disability and HC. Higher sensorimotor connectivity has been found previously in early MS patients with either no or minimal disability,^6,7^ frequently interpreted as beneficial functional reorganization to limit clinical impairment although this remains to be proven.^26^ In later stages of MS, increased sensorimotor connectivity has also been observed previously but without clinical relations.^8,10^ In our study, no FC changes were observed in patients with low disability, while patients with more severe disability only showed increased FC compared to controls. Decreased connectivity was not seen in our data, but has been observed previously, related to worse disability.^11,13^ It was previously suggested that a decrease in connectivity could follow from an initial increase in FC, which remains difficult to prove given the strong lack of longitudinal data.

Our findings indicate that increases in connectivity that are sufficient to alter global motor network efficiency might actually not be related to favourable disability outcomes at all. This is supported by previous studies demonstrating association between increased connectivity and worse cognitive functioning,^27^ and disability.^28^ In our study, S1 also showed higher FC with several sensorimotor regions, which might be driven by a disruption of mechanisms designed to guide meaningful ascending and/or descending sensorimotor information due to pathological processes in the corticospinal tract. This disruption could result in a loss of inhibition of input reaching S1, resulting in higher connectivity, despite the fact that the information contained within these signals could essentially be noise. However, such causal claims remain speculative, as the opposite may also be true, that is, that the efficiency and connectivity change could reflect a beneficial mechanism present in patients with severe damage only. As our analyses are limited to cross-sectional, non-directional connectivity measures, future studies should pinpoint how such network changes come to be. The use of empirical computational models might give insight into the change and interaction of functional and structural processes over time.^29^

### Structure and function

Our study showed that functional measures provide added value beyond structural damage. Previous studies have shown that higher levels of disability correlate moderately to white matter damage, measured by lesion load.^2^ Recently, structural network efficiency was shown to explain 58% of disability variation, much more than simply averaging damage.^24^ In addition, using an empirical informed model, WM damage in the form of loss of diffusion-based tracts was shown to drive increased connectivity and network efficiency changes.^29^ As such, it would be of high interest to combine structural and functional network measures in the future.

GM damage has also been related to disability, especially DGM and thalamic atrophy.^30^ The thalamus has gained considerable interest in MS research as atrophy may precede clinical symptoms,^31^ and thalamic atrophy and function strongly correlate to both cognitive decline^27^ and disability progression.^5,32^ While DGM volume was also a significant correlate of disability in our model, we did not observe significant functional efficiency changes in the thalamus, which is in line with a previous study showing no correlation between thalamic atrophy and thalamic connectivity.^27^ We did find an increased efficiency of the pallidum and S1, both areas strongly connected to the thalamus and important in the regulation of movement and sensory processing. The pallidum as a correlate of disability was supported by another recent study.^33^ Together, these studies highlight the added value of regional and network-based information.

### Future directions

We included areas beyond ‘typical’ motor regions such as M1 and S1, based on a previous study,^24^ but not cognitive regions that could still influence disability or cerebellar subregions. Furthermore, we used relative FC scores due to inter-participant variabilities that warrant some caution.^23^ How best to correct for this variability requires future studies to determine. Even though a recent study showed that cerebral network changes can explain disability beyond spinal cord atrophy,^24^ the latter was not included in our study. Furthermore, other graph analytical concepts such as network centrality could provide additional information. Including neurological tests such as the nine-hole peg test could detect other aspects of sensorimotor dysfunction, enabling a more comprehensive way of defining disability. Finally, our approach was based on commonly used ‘static’ connectivity, that is, efficiency across the entire scan, but unique information may reside in dynamic fluctuations and stability of connectivity patterns.^34,35^ Finally, future multimodal longitudinal studies are needed to investigate the order of events leading to the accumulation of disability.

## Conclusion

Using an advanced network imaging approach, this study shows that functional changes in the SMN centred around S1 are associated with high disability, independently from structural damage. Patients with severe disability (aid or assistance required to walk) showed increased local efficiency and connectivity of S1, suggesting that increases in brain network efficiency may be a marker of poorer clinical function.

## Supplemental Material

MSJ966292_Supplementary_Figure_1 – Supplemental material for Increased functional sensorimotor network efficiency relates to disability in multiple sclerosis Click here for additional data file. Supplemental material, MSJ966292_Supplementary_Figure_1 for Increased functional sensorimotor network efficiency relates to disability in multiple sclerosis by Myrte Strik, Declan T Chard, Iris Dekker, Kim A Meijer, Anand JC Eijlers, Matteo Pardini, Bernard MJ Uitdehaag, Scott C Kolbe, Jeroen JG Geurts and Menno M Schoonheim in Multiple Sclerosis Journal
