# Supplementary material for: Increased functional sensorimotor network efficiency relates to disability in multiple sclerosis
Source: Mult Scler. 2020 Oct 26;27(9):1364–73. doi: 10.1177/1352458520966292 (PMC8358536; doi:10.1177/1352458520966292)
Supplement: MSJ966292_Supplementary_Figure_1 – Supplemental material for Increased functional sensorimotor network efficiency relates to disability in multiple sclerosis [file MSJ966292_Supplementary_Figure_1.pdf]

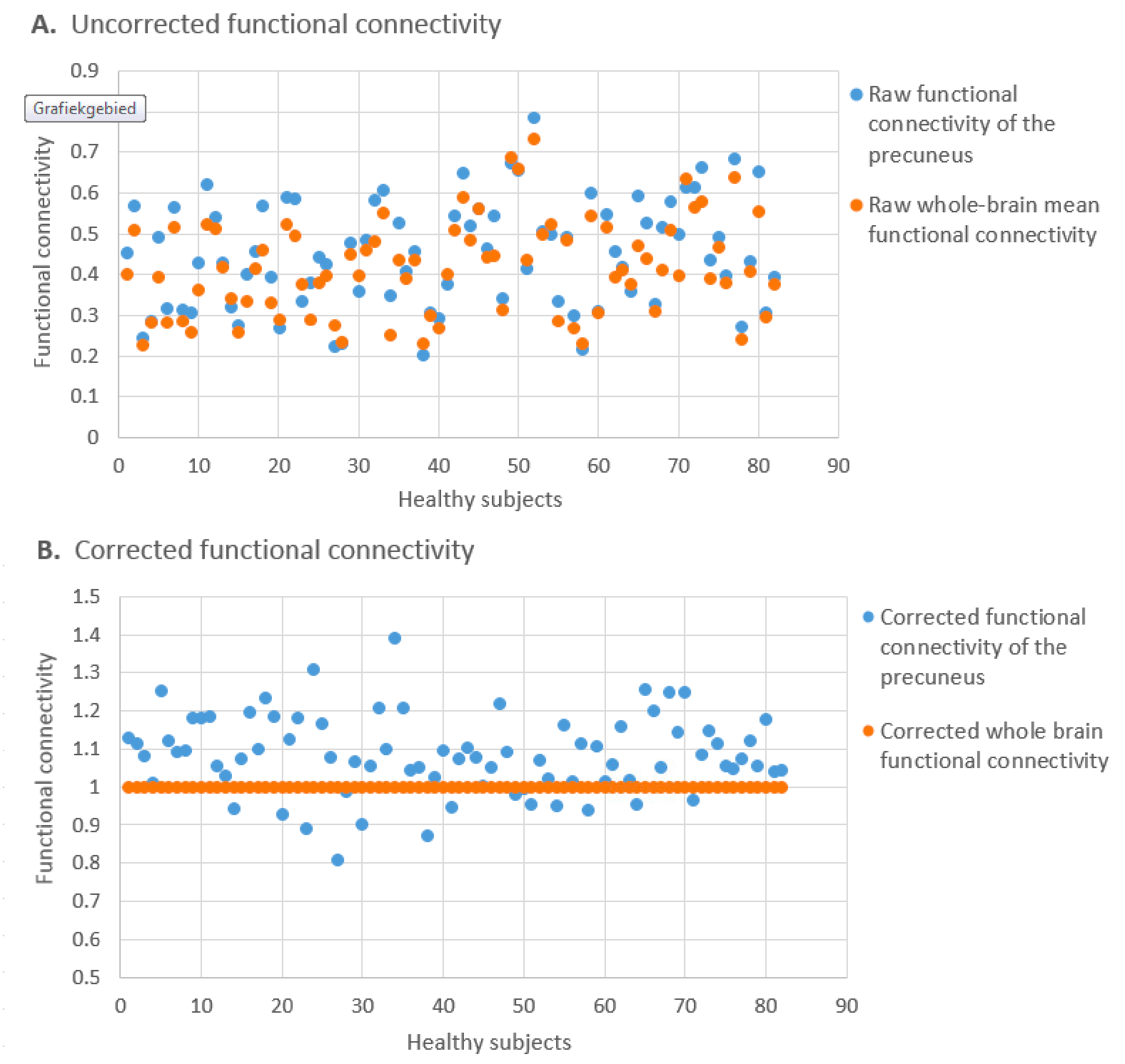

Supplementary Figure 1. Inter-subject functional connectivity variability. A) To visualize the variability in functional connectivity (FC) profiles between subjects, for each healthy subject (x-axis) the average whole-brain FC (orange) and the uncorrected FC values (blue) of the precuneus, a major hub in the brain, are plotted on the y-axis. This plot suggests that the precuneus is not a major hub as it is often not higher than the average connectivity of the population. However, compared to each individual whole brain FC, the FC of the precunues is usually higher. B) By dividing each connection by each individual average whole-brain FC, the variability of the hubness of the precuneus is less and can be more readily compared between subjects and groups.
